# Selective expression of sense and antisense transcripts of the sushi-ichi-related retrotransposon – derived family during mouse placentogenesis

**DOI:** 10.1186/s12977-015-0138-8

**Published:** 2015-02-03

**Authors:** Christine Henke, Pamela L Strissel, Maria-Theresa Schubert, Megan Mitchell, Claus C Stolt, Florian Faschingbauer, Matthias W Beckmann, Reiner Strick

**Affiliations:** Department of Gynaecology and Obstetrics, Laboratory for Molecular Medicine, Friedrich-Alexander University Erlangen-Nürnberg (FAU), University-Clinic Erlangen, Erlangen, Germany; Institute of Biochemistry, D-91054 Erlangen, Germany

**Keywords:** Retrotransposon, Mart, SIRH, Mouse placenta, Trophoblast, Antisense transcripts, TASA-TD

## Abstract

**Background:**

LTR-retrotransposons became functional neogenes through evolution by acquiring promoter sequences, regulatory elements and sequence modification. Mammalian retrotransposon transcripts (*Mart1-9*), also called *sushi-ichi-*related retrotransposon-homolog (*SIRH*) genes, are a class of Ty3/gypsy LTR-retroelements showing moderate homology to the *sushi-ichi* LTR-retrotransposon in pufferfish. *Rtl1/Mart1* and *Peg10/Mart2* expression in mouse placenta and demonstration of their functional roles during placental development exemplifies their importance in cellular processes. In this study, we analyzed all eleven mouse *Mart* genes from the blastocyst stage and throughout placentogenesis in order to gain information about their expression and regulation.

**Results:**

Quantitative PCR, *in situ* hybridization (ISH) and immunoblotting showed various expression patterns of the 11 mouse *Mart* genes through different placental stages. Zcchc5/*Mart3, Zcchc16/ Mart4 and Rgag1/Mart9* expression was undetectable. *Rtl1/Mart1, Peg10/Mart2, Rgag4/Mart5 – Cxx1a,b,c/Mart8b,c,a* gene expression was very low at the blastocyst stage. Later placental stages showed an increase of expression for *Rtl1/Mart1, Rgag4/Mart5 – Cxx1a,b,c/Mart8b,c,a,* the latter up to 1,489 molecules/ng cDNA at E9.5. From our recently published findings *Peg10/Mart2* was the most highly expressed *Mart* gene. ISH demonstrated sense and antisense transcript co-localization of *Rgag4/Mart5* to *Cxx1a,b,c/Mart8b,c,a* in trophoblast subtypes at the junctional zone, with an accumulation of antisense transcripts in the nuclei. To validate these results, we developed a TAG-aided sense/antisense transcript detection (TASA-TD) method, which verified sense and antisense transcripts for *Rtl1/Mart1, Rgag4/Mart5 – Cxx1a,b,c/Mart8b,c,a*. Except for *Rtl1/Mart1* and *Cxx1a,b/Mart8b,c* all other *Mart* genes showed a reduced amount of antisense transcripts. Northern blot and 5′ and 3′ RACE confirmed both sense and antisense transcripts for *Ldoc1/Mart7* and *Cxx1a,b,c/Mart8b,c,a*. Immunoblotting demonstrated a single protein throughout all placental stages for Ldoc1/Mart7, but for Cxx1a,b,c/Mart8b,c,a a switch occurred from a 57 kDa protein at E10.5 and E14.5 to a 25 kDa protein at E16.5 and E18.5.

**Conclusions:**

RNA and protein detection of mouse *Mart* genes support neo-functionalization of retrotransposons in mammalian genomes. Undetectable expression of *Zcchc5/Mart3, Zcchc16/Mart4* and *Rgag1/Mart9* indicate no role during mouse placentogenesis. *Rgag4/Mart5* to *Cxx1a,b,c/Mart8b,c,a* gene expression support a role for differentiation from the ectoplacental cone. *Mart* antisense transcripts and protein alterations predict unique and complex molecular regulation in a time directed manner throughout mouse placentogenesis.

**Electronic supplementary material:**

The online version of this article (doi:10.1186/s12977-015-0138-8) contains supplementary material, which is available to authorized users.

## Background

Transposable elements are found from bacteria to humans and contribute to the dynamics of genomes, where they profoundly alter and affect structure and function. Mammals in particular have an abundant amount of transposable elements, which originated from retroelements (also called retrotransposons) integrating into the genome over time. In the murine genome 40% of sequences account for transposable elements [1]. Many protein coding retroelements, like the human endogenous retroviruses (ERV) belonging to the long terminal repeat (LTR) retrotransposons, are known. The class of Metaviridae or Ty3/gypsy LTR retroelements is classified into three genera according to the presence of the *env* gene and chromodomain [2]. Furthermore, *Metaviridae* derived genes in the human genome have been classified into five families [2]. One of them is *sushi*, an LTR retrotransposon identified in the pufferfish *Takifufu rubripies* and other fishes [3]. The *sushi-ichi* gene represents a full length retrotransposon from this family. Functional *sushi* retrotransposons were not found in mammals, only *sushi* related neogenes. *Ma*mmalian *r*etrotransposon *t*ranscripts (*Mart*) (also called *s*ushi-*i*chi-related *r*etrotransposon *h*omologs (*SIRH*)) are derived from the *gag* genes of Ty3/gypsy LTR retroelements [4-11] and identified in eutherian mammals, but also in marsupials [12].

**Figure 1:**
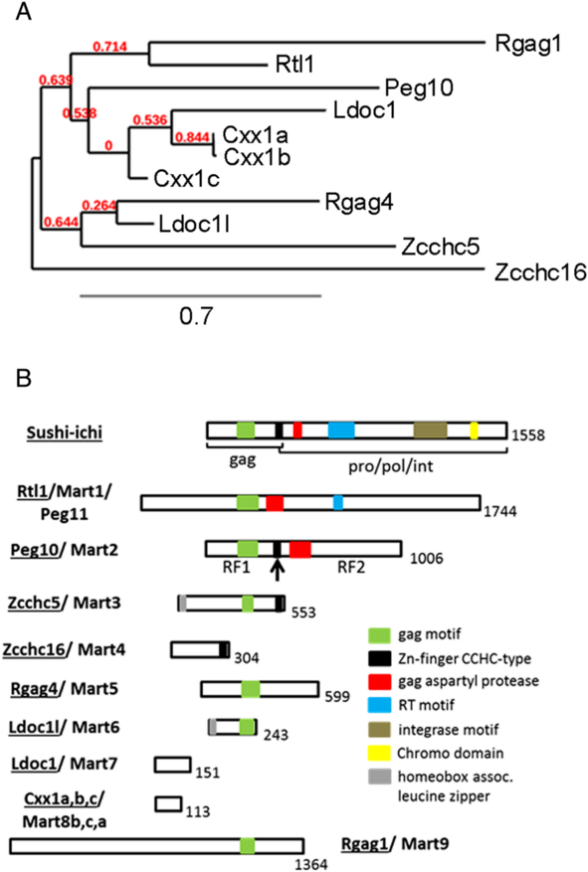
**Phylogenetic tree and schematic drawing of the gene structure of the** ***sushi-ichi*** **retrotransposon gene and the mouse** ***Mart*** **genes. A)** Phylogenetic and DNA sequence analysis indicates that the *Mart* gene family originated independently several times in the ancestors of placentalia. Long distances of the lines between single genes represent greater DNA differences, for example *Zcchc16/Mart4* is considered an outgroup from all other *Mart* genes. On the other hand, *Cxx1a,b/Mart8b,c* belong to one gene clade, whereas *Cxx1c/Mart8a* is on a different gene clade, which is ancestral to *Cxx1a /Mart8b* and *Cxx1b/Mart8c*. Therefore *Cxx1c/Mart8a* could be the first evolutionary *Cxx1/Mart8* gene integrated into the genome, whereas *Cxx1a/Mart8b* and *Cxx1b/Mart8c* later duplicated events. **B)** Listed is the first founded *sushi-ichi* retrotransposon from *Puffer fish* and then the eleven mouse *Mart* genes. Numbers indicate amino acid lengths of proteins and colored boxes symbolize the different protein motifs identified as the gag (group specific antigen), pro (protease), pol (polymerase) and int (integrase). The arrow on the Peg10/Mart2 protein points to the −1 frame shift, which results in two open reading frames (RF1 and RF2).

One hypothesis states that through evolution frequent independent domestications occurred in ancestors of placentalia and that re-activation and gene duplications of these Metaviridae led to the highest number of conserved retroelement-derived domesticated genes [2,13]. This conservation argues for essential functions especially during placentogenesis. Some examples are the human *ERV* gene family and the eleven mouse *Mart* genes with high sequence homology. Figure 1A represents a phylogenetic tree, showing the inferred evolutionary relationships between the single *Mart* genes [2]. All eleven *Mart* genes (*Mart 1*–*9)* represent single gene copies, including three single gene copies of *Cxx1/Mart8* (*Cxx1a,b,c/Mart8b,c,a*) in both mouse and human [14]. Figure 1B shows the first analyzed retrotransposon “*sushi-ichi*” and the mouse *Mart* genes with their main motives. Except for *Rtl1/Mart1*, *Peg10/Mart2* and *Ldoc1l/Mart6* all other *Mart*-genes are located on chromosome X. To date all *Marts* have newly recruited promoter sequences with no LTRs [2] and deletions which have occurred over time mostly within the pol region (Figure 1B) [14]. This gene structure indicates that the *Mart* genes are not functional autonomous retrotransposons [11,14]. Importantly, three *Mart* genes contain the CCHC-type Zn-finger (consensus: RK*C*YN*C*GKPG*H*MARD*C*PE), a motif which was shown in retroviral nucleocapsid proteins to help in viral genome packaging, but was also found in eukaryotic proteins involved in RNA or ssDNA binding [15] (Figure 1B). *Cxx1a,b,c/Mart8b,c,a* are flanked by regions of similar sequences indicating that their gene structure was formed by duplication events during evolution [14]. Two paternally expressed genes (*Peg*), *Rtl1/Mart1* (*Peg11*) and *Peg10/Mart2* are maternally imprinted. Mouse *Rtl1/Mart1* is localized on chromosome 12, whereas *Peg10/Mart2* is on chromosome 6. Additionally, the maternal chromosome 12 expresses an antisense transcript of *Rtl1/Mart1* (*Anti-Peg11*) [16]. *Rtl1/Mart1* was shown to be expressed in the human and murine placenta [17,18]. Knockout mice showed an essential function between the mid and terminal stages of placenta development. One of the best characterized genes is *Peg10*/*Mart2*, where a −1 frame shift resulted in two gag-/pol-like *open reading frames* (ORF) with an identical N-terminus (Figure 1B) [7,19,20]. Expression of human *PEG10/MART2* was detected during embryonic development, as well as in placenta, brain, lung, testes and ovary [7,20]. Mouse *Peg10/Mart2* was shown in embryonic tissue and in different trophoblast subtypes of the placenta [6,21,22]. For example, *Peg10/Mart2* RNA specifically localized to the parietal trophoblast giant cells (pTGC) in the junctional zone and sinusoidal trophoblast giant cells (sTGC) of the labyrinth layer [21]. *Peg10/Mart2* knockout mice had smaller than normal placentas, absence of spongiotrophoblast markers and embryonic lethality until E10.5 [22,23]. Lastly the human *LDOC1/MART7* gene was found ubiquitously expressed in human tissues and down regulated in some cancer cell lines [8].

The mammalian placenta is formed during embryonic development by maternal and fetal cells. It is essential for embryo survival, to transport nutrients and waste products and is important for hormone production during gestation [24,25]. The mouse placenta has three additional layers compared to the human placenta (trichorial): decidua, junctional zone and the labyrinth formed by different trophoblast subtypes. During development the inner cell mass (ICM) and trophoectoderm of the blastocyst differentiates into four main trophoblast subtypes: trophoblast giant cells (TGCs), spongiotrophoblasts (STs), glycogen trophoblasts (GlyT) and multinucleated syncytiotrophoblasts (SCTs) [24,26,27]. There are also different subtypes of TGCs with different origins. Primary TGCs are derived from the mural trophoectoderm however, they also can stem from extraembryonic ectoderm, the placental cone or STs [27,28]. TGCs are also involved in blastocyst attachment in early embryogenesis and exhibit an invasive behavior into the uterine wall after implantation [29]. The junctional zone of the murine placenta is formed adjacent to TGCs by STs and GlyTs. STs and GlyTs are derived from progenitors in the ectoplacental cone and also represent progenitor cells themselves [26]. STs can differentiate into TGCs and into GlyTs after E12.5 and become polyploid through endoreduplication [26,30]. After differentiation, several subtypes of TGCs are found in the junctional zone and labyrinth layer. Distinctively, pTGCs are unusually large, are highly polyploid and traverse the junctional zone [31]. The main function of TGCs is the production of hormones, for example prolactin and placental lactogens [32,33]. STs are also able to migrate in the decidua layer as well as in the labyrinth and are the main source of Igf2 production in the second half of gestation [33,34]. The labyrinth layer is formed by three trophoblast subtypes: the mononucleated sTGC and two multinucleated SCTs (SCT-I, SCT-II), which differentiates from the extraembryonic ectoderm and are responsible for the nutrient and gas exchange between mother and fetus [26]. STGC surround the maternal blood vessels and are adjacent to the multinucleated SCTs in the labyrinth layer. Canal and spiral artery associated TGCs are also located in the maternal blood system but more upstream in the canal spaces and spiral arteries [28].

Although mouse *Rtl1*/*Mart1* to *Ldoc1l/Mart6* genes were found expressed in most organs tested during embryonic development [14], to date their expression pattern in mouse placenta is unknown except for the characterized *Rtl1/Mart1* and *Peg10/Mart2,* and recently for *Ldoc1/Mart7* [17,21,22,35]. To gain further knowledge of the *Mart* gene family, we performed gene expression quantification (qPCR) of the *Mart* family from mouse blastocysts (E4.5) and placentae stages E8.5 to E18.5 to determine their expression throughout placentogenesis. Cellular localization of expressed *Mart* genes was analyzed by *In situ* hybridization (ISH) at different stages (E8.5, E14.5/E15.5), as well as protein expression of Ldoc1/Mart7 and Cxx1/Mart8 by immunoblotting (E10.5, E14.5, E16.5, E18.5). Both ISH and a first strand cDNA analysis showed the presence of antisense transcripts supporting RNA/protein regulation in the cell.

## Results

### Mouse *Mart* genes showed differential RNA expression at the blastocyst stage and throughout placentogenesis especially localizing to trophoblasts in the junctional zone

**Figure 2:**
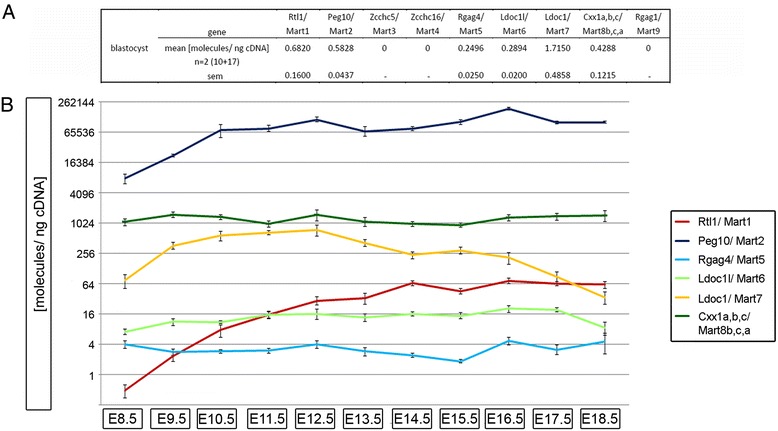
**Gene expression of mouse** ***Mart*** **genes in blastocysts and placentae during embryonic development from E8.5 to E18.5. (A)** Mean expression of *Rtl1/Mart1* to *Rgag1/Mart9* in molecules per ng cDNA (molecules/ng cDNA) ± sem from 2 pooled samples of 10 and 17 blastocysts (E4.5). **(B)** Line diagram represents a logarithmic scale of the mean expression of *Rtl1/Mart1*, *Peg10/Mart2*, *Rgag4/Mart5*, *Ldoc1l/Mart6*, *Ldoc1/Mart7* and *Cxx1a,b,c/Mart8b,c,a* in molecules per ng cDNA (molecules/ng cDNA) ± sem from n = 4 to n = 9 placentae for embryonic days E8.5 to E18.5. Congruent with blastocyst expression in 2**A**, the *Zcchc5/Mart3*, *Zcchc16/Mart4* and *Rgag1/Mart9* genes showed no expression from E8.5 and E18.5 placenta (data not shown). All absolute gene expression values are listed in Additional file 3: Table S3.

In order to determine the expression level of the mouse *Mart* genes we performed absolute qPCR from two pooled samples of blastocysts (E4.5), one containing 10 and the other with 17 blastocysts. We then analyzed *Rtl1/Mart1* and *Zcchc5/Mart3 to Rgag1/Mart9* RNA expression with eleven different embryonic stages of placentae from E8.5 to E18.5. Furthermore we performed ISH with the four highly expressed *Mart* genes *Rgag4/Mart5*, *Ldoc1l/Mart6, Ldoc1/Mart7* and *Cxx1a,b,c/Mart8b,c,a*, using placental tissues from an earlier stage E8.5 and a later stage E14.5/E15.5, where all trophoblast subtypes are formed. The expression of all *Mart* genes in blastocysts was either very low or un-detectable. For example, we detected mean values of 0.68 molecules/ng cDNA for *Rtl1/Mart1* and 1.71 molecules/ng cDNA for *Ldoc1*/*Mart7*; whereas RNA amounts for *Zcchc5/Mart3*, *Zcchc16/Mart4* and *Rgag1/Mart9* were un-detectable (Figure 2A). Regarding *Mart* expression during placentogenesis, we compared our previously published results of *Peg10/Mart2* with all other *Mart* genes in this study (Figure 2B). Interestingly, *Peg10/Mart2* was the highest expressed gene throughout all placental stages, as previously analyzed [21]. *Rtl1/Mart1* RNA expression levels were maximal at E14.5 with a mean of 64.38 molecules/ng cDNA and represented the fourth highest *Mart* gene during placentogenesis (Figure 2B). Similar to blastocysts *Zcchc5/Mart3*, *Zcchc16/Mart4* and *Rgag1/Mart9* were also found un-detectable throughout placentogenesis (data not shown).

**Figure 3:**
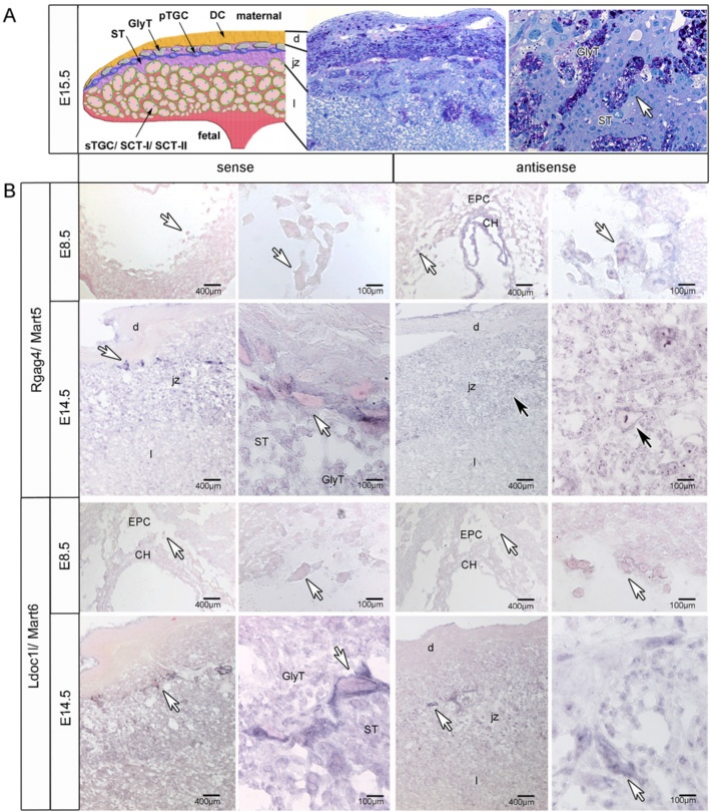
**Localization of** ***Rgag4/Mart5*** **and** ***Ldoc1l/Mart6*** **in the mouse placenta at E8.5 and E14.5 by ISH. (A)** Schematic drawing shows an overview of the structure of the mouse placenta at E15.5 with decidua cells (DC) in the decidua (d); junctional zone (jz) containing parietal trophoblast giant cells (pTGC); glycogen trophoblasts (GlyT) and spongiotrophoblasts (ST) and the labyrinth (l) containing sinusoidal trophoblast giant cells (sTGC) and syncytiotrophoblast-I/-II (SCT-I, SCT-II). Two right pictures show Periodic acid-Shiff (PAS) stained placenta tissue sections congruent with tissue layers and cells identified in the left drawing. Darkly stained purple PAS positive cells identify glycogen trophoblasts (GlyT). Also shown are negatively stained spongiotrophoblasts (ST) and parietal trophoblast giant cells (white arrow). **(B)** *In situ* hybridization identifying *Rgag4/Mart5* and *Ldoc1l/Mart6* sense and antisense transcripts at E8.5 and E14.5. For each transcript detected, the left and right panels with black arrows pointing to pTGC identify low and high magnifications of the same regions. White arrows show pTGC in different magnified regions of the same tissue. Bars are shown in μm. Decidua (d), junctional zone (jz), labryrinth (l), EPC, ectoplacental cone; CH, chorion; spongiotrophoblast (ST); glycogen trophoblast (GlyT).

*Rgag4/Mart5* and *Ldoc1l/Mart6* were the lowest expressed genes among the *Marts* (Figure 2B). ISH using specific probes for sense and antisense RNA showed expression of both *Rgag4/Mart5* transcripts (Figure 3B). For example, at E14.5 *Rgag4*/*Mart5* sense RNA localized to the placental junctional zone with the strongest signal in the cytosol of pTGCs. This was in contrast to E8.5 where no expression of *Rgag4/Mart5* sense transcripts was detected. Additionally, *Rgag4*/*Mart5* was also expressed in STs and GlyTs of the junctional zone (Figure 3B). To verify the localization of *Rgag4/Mart5* expression in STs and GlyTs we performed PAS-staining on paraffin sections of E15.5 placentas and identified GlyTs ‘islands’ enriched for glycogen in the junctional zone (Figure 3A). Importantly, both STs and GlyTs showed a similar *Rgag4/Mart5* expression level. On the other hand the labyrinth layer as well as the decidua showed no expression of *Rgag4/Mart5* at E14.5 (Figure 3B). *Rgag4/Mart5* antisense transcripts were localized throughout the whole junctional zone of E14.5 placenta but in contrast to the sense transcript, it was detected in the nuclei of pTGCs at E8.5 and E14.5. Regarding the *Ldoc1l/Mart6* gene, expression was similar to *Rgag4/Mart5* (Figure 2B). Detection of transcripts at E14.5 with ISH also showed sense and antisense transcripts in the junctional zone with the highest expression in pTGCs. The *Ldoc1l/Mart6* antisense transcript also co-localized to the nuclei, whereas the sense transcript was more localized to the cytosol (Figure 3B).

**Figure 4:**
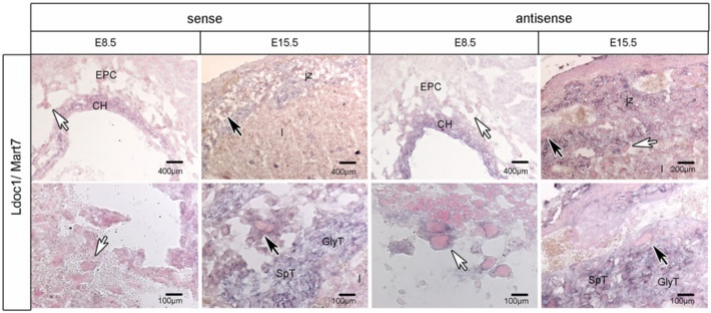
**Localization of** ***Ldoc1/Mart7*** **in the mouse placenta at E8.5 and E15.5 by ISH.** *Ldoc1/Mart7* sense and antisense transcripts are shown in placental tissues at E8.5 and E15.5. Lower magnifications of tissue sections are shown in upper panels and higher magnifications of specific regions in lower panels. Black arrows identify positive pTGCs from congruent low (top) and high (bottom) magnified regions in both pictures, whereas white arrows identify pTGCs from new tissue regions. Bars are shown in μm. For all abbreviations see Figure 3.

**Figure 5:**
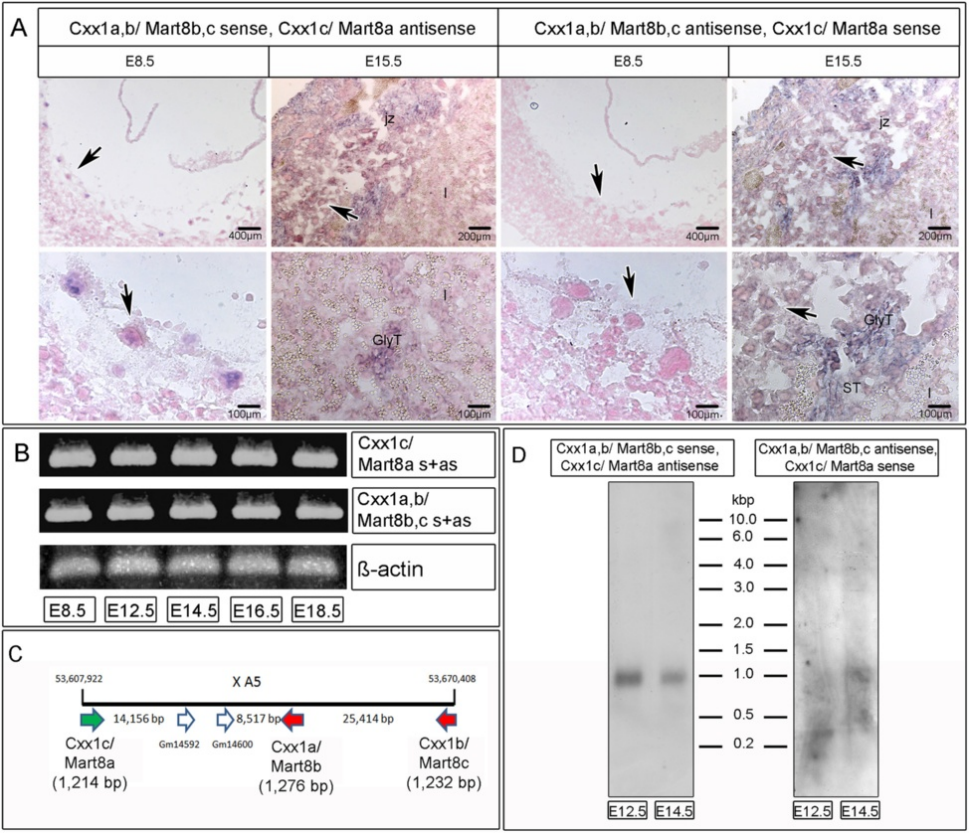
**ISH localization at E8.5 and E15.5 and PCR of the three different** ***Mart8a, −b*** **and** ***-c*** **gene loci in mouse placenta. (A)** ISH identifying *Cxx1c/Mart8a* antisense and *Cxx1a,b/Mart8b,c* sense transcripts (left panels) and *Cxx1b/Mart8a* sense and *Cxx1a,b/Mart8b,c* antisense transcripts (right panels) at E8.5 and E15.5. Black arrows identify positive pTGCs from congruent low (top) and high (bottom) magnified regions in both pictures. Bars are shown in μm. For abbreviations see Figure 3. **(B)** PCR of *Cxx1c/Mart8a* and *Cxx1a,b/Mart8b,c* sense (s) and anti-sense (as)transcripts at E8.5, E12.5, E14.5, E16.5 and E18.5 using specific primers with placental cDNA. *Beta-actin* was used as positive control. **(C)** Schematic drawing of the mouse *Cxx1/Mart8* genes and two additional genes Gm14592, Gm14600 on chromosome X A5 according to the current assembly: GRCm38.p2. *Cxx1a/Mart8b* and *Cxx1b/Mart8c* (red arrows) are localized on the opposite DNA strand thus, in the opposite orientation in context to *Cxx1c/Mart8a* (green arrow). All DNA regions and the length of each *Mart* gene are signified in base pairs. **(D)** Northern analyses identifying *Cxx1c/Mart8a* antisense and *Cxx1a,b/Mart8b,c* sense transcripts (left lanes) and *Cxx1b/Mart8a* sense and *Cxx1a,b/Mart8b,c* antisense transcripts (right lanes) at E12.5 and E14.5.

QPCR of *Ldoc1/Mart7* and *Cxx1a,b,c/Mart8b,c,a* demonstrated the third and second highest expression levels, respectively, of all *Mart* genes. For *Ldoc1/Mart7* an increase of gene expression occurred from E8.5 (mean: 73.50 molecules/ng cDNA) to E12.5 (mean: 737.16 molecules/ng cDNA), which was followed by a significant decrease until E18.5 (Figure 2B). ISH of *Ldoc1/Mart7* sense and antisense transcripts at E8.5 and E15.5 showed a weaker expression of both transcripts in pTGCs, chorion and EPC at E8.5, but a strong cytosolic expression in pTGCs, STs and GlyTs at E15.5 (Figure 4). No expression of *Ldoc1/Mart7* was found in trophoblasts of the labyrinth layer at E15.5 (Figure 4). Due to over 81% DNA sequence identity between the *Cxx1a/Mart8b*, *Cxx1b/Mart8c* and *Cxx1c/Mart8a* genes, one primer set was designed to identify expression levels for all three genes by qPCR. Additionally, *Cxx1a/Mart8b* and *Cxx1b/Mart8c* showed over 99% in DNA sequence identity. The localization, orientation and the length of all three genes on chromosome X are represented in Figure 5C. The expression of *Cxx1a,b,c/Mart8b,c,a* was higher and more constant between E8.5 (mean: 1,061.00 molecules/ng cDNA) and E18.5 (mean: 1,431.50 molecules/ng cDNA) compared to *Ldoc1/Mart7* (Figure 2). Considering that *Cxx1a/Mart8b* and *Cxx1b/Mart8c* are located on the opposite strand in context to *Cxx1c/Mart8a* (Figure 5C), hybridization of the *Cxx1a/Mart8b* antisense probe with placental tissues (E8.5, E15.5), could identify *Cxx1c/Mart8a* antisense and *Cxx1a,b/Mart8b,c* sense transcripts (Figure 5A left). On the other hand hybridization of a *Cxx1a/Mart8b* sense probe, localized simultaneously *Cxx1c/Mart8a* sense and *Cxx1a,b/Mart8b,c* antisense transcripts (Figure 5A right). Results showed *Cxx1c/Mart8a* antisense and *Cxx1a,b/Mart8b,c* sense expression in pTGCs nuclei at E8.5, whereas *Cxx1c/Mart8a* sense and *Cxx1a,b/Mart8b*,*c* antisense expression was only weakly detected around the nuclei of pTGCs at E8.5. At E15.5 the expression of all three *Cxx1/Mart8* genes were localized in the pTGCs, STs and GlyTs in the junctional zone (Figure 5A) and in the GlyT ‘islands’ of the labyrinth layer (Figure 5A). In addition, using PCR we analyzed *Cxx1c/Mart8a* and *Cxx1a,b/Mart8b,c* expression of placenta cDNA from different stages (E8.5, E12.5, E14.5, E16.5 and E18.5) with gene specific primers, but with no distinction between sense and antisense transcripts. Results showed no differences in *Cxx1c/Mart8a* and *Cxx1a,b/Mart8b,c* expression between the different placental stages, which was comparable to our qPCR findings (Figure 2B).

### Quantification of *Mart* gene sense and antisense transcripts demonstrated different expression levels

**Figure 6:**
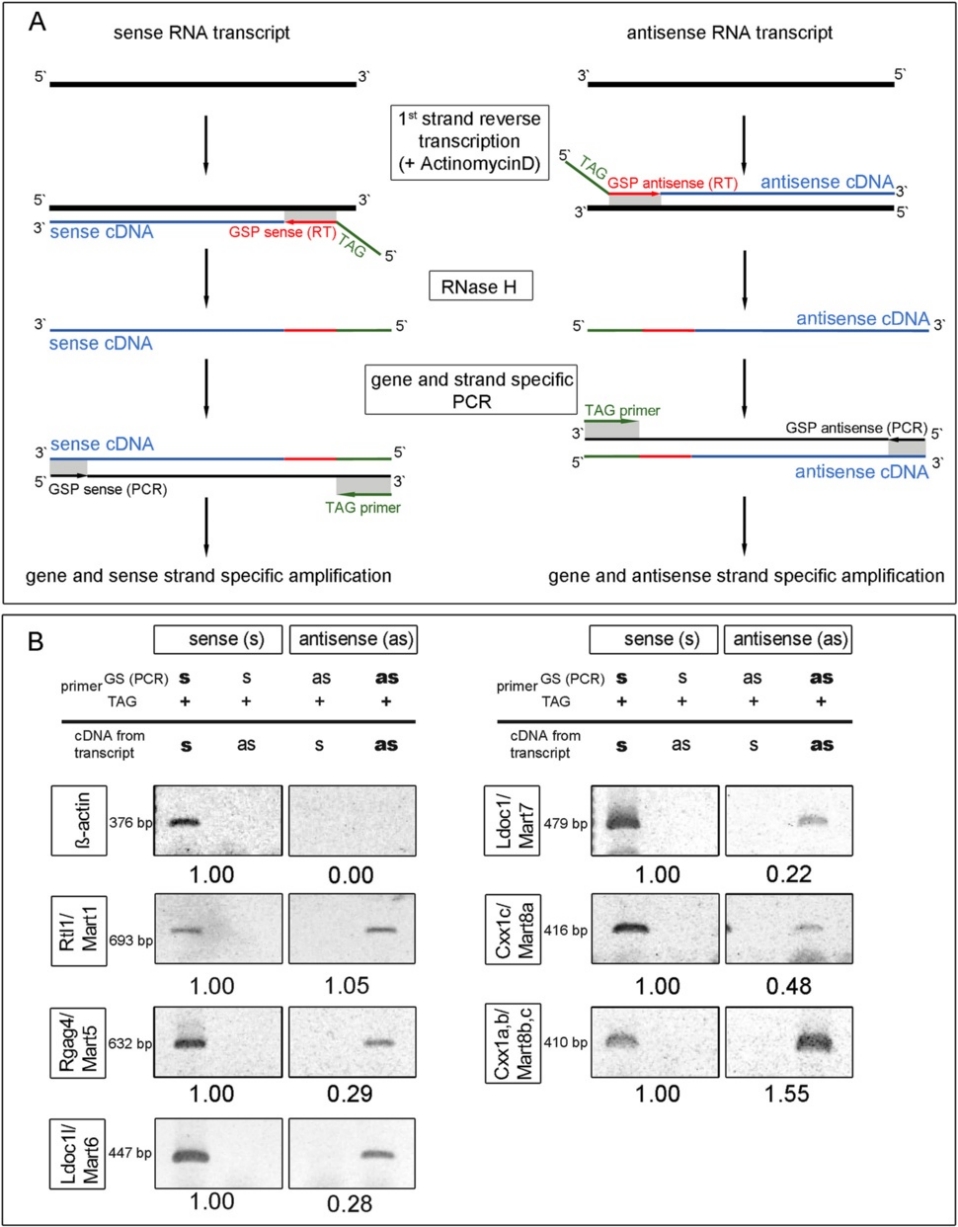
**Sense and antisense transcripts of mouse** ***Rtl1/Mart1*** **,** ***Rgag4/Mart5*** **,** ***Ldoc1l/Mart6*** **,** ***Ldoc1/Mart7*** **and** ***Cxx1a,b,c/Mart8b,c,a*** **in placenta tissue at E14.5 using TAG-aided sense/antisense transcript detection (TASA-TD). (A)** Schematic drawing of the methodology used for first strand cDNA synthesis with a gene and strand specific TAG labeled primer (GSP sense/antisense (RT) TAG) and the following PCR with another gene and strand specific primer (GSP sense/antisense (PCR)) and a TAG-primer) **(B)** First strand cDNA synthesis and PCR identification as well as quantification of sense (s) and antisense (as) transcripts of *Rtl1/Mart1*, *Rgag4/Mart5*, *Ldoc1l/Mart6*, *Ldoc1/Mart7*, *Cxx1c/Mart8a* and *Cxx1a,b/Mart8b,c. Beta-actin* was used as control.

**Figure 7:**
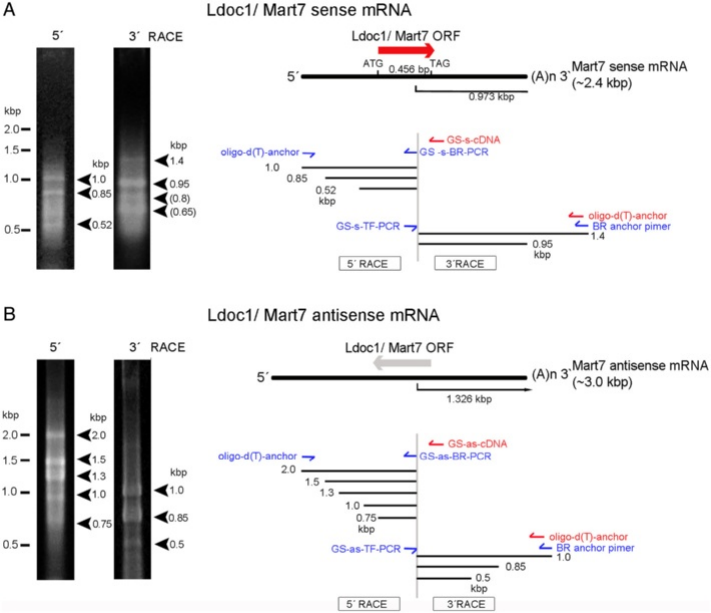
**Determination of** ***Ldoc1/Mart7*** **sense and antisense transcripts using 5′ and 3′ RACE. A)** 5′ and 3′ RACE for sense transcripts were performed with a gene specific (GS) sense (s) primer for first strand cDNA synthesis (cDNA) and subsequent PCR. PCR products were analyzed on agarose gels with ethidium bromide, which identified all extended transcripts (left) and are shown in the gene map (right). **B)** 5′ and 3′ RACE for antisense transcripts were performed with a gene specific (GS) antisense (as) primer for first strand cDNA synthesis (cDNA) and subsequent PCR. PCR products showed all extended transcripts (left) and in the gene map (right).

To corroborate our ISH results we further analyzed RNA expression of both sense and antisense transcripts for the *Mart* genes using first strand cDNA synthesis and then gene and strand specific PCR. Due to the fact that RNA transcripts and especially transcripts of retrotransposon derived genes form secondary structures and hairpins, false positive results can occur through self-priming events (data not shown). To circumvent this problem we developed a new technique called “TAG-aided sense/antisense transcript detection” (TASA-TD), involving a specific TAG-sequence not present in the mouse genome, which was located at the 5′ ends of each annealing sense or antisense primer (Figure 6A, Additional file 1: Table S1). Initially, first strand cDNA synthesis was performed using the gene specific primers fused with the TAG-sequence (GSP sense/antisense (RT) TAG). The addition of Actinomycin D prevented false positive results by halting the minus strand transfer during reverse transcription [36]. Gene and strand specific PCR was then performed using two primers to amplify each transcript: a GSP sense/antisense (PCR) and a specific primer unique only for the TAG-sequence. In this way amplification was specific for cDNA with a TAG-sequence overhang (Figure 6A). *Rtl1/Mart1* (*Peg11*) was previously demonstrated to have an antisense transcript, thus represented a positive control [16] (Figure 6B). We detected similar amounts of *Rtl1/Mart1* sense and antisense transcripts (1: 1.05), whereas *Rgag4/Mart5, Ldoc1l/Mart6* and *Ldoc1/Mart7* showed 3.5-4.5-fold less antisense than sense transcripts at E14.5. Interestingly, *Cxx1c/Mart8a* antisense transcript was 2.1-fold less, but in contrast *Cxx1a,b/Mart8b,c* antisense transcripts were 1.5-fold higher expressed than the sense transcripts. To further confirm sense and antisense transcripts, we performed Northern blot analyses and 5′ and 3′ RACE to determine *Cxx1/Mart8* and *Ldoc1/Mart7* transcript sizes. Due to the fact that *Cxx1a,b,c/Mart8b,c,a* are highly homologues to each other and *Cxx1a/Mart8b* and *Cxx1b/Mart8c* are transcribed complementary to *Cxx1c/Mart8a*, it was not possible to discriminate the *Cxx1/Mart8* sense and antisense transcripts separately using probes (Figure 5B, C). For these reasons we performed Northern blot analysis, which identified ~1.0 kb transcripts of *Cxx1c/Mart8a* antisense and *Cxx1a,b/Mart8b,c* sense at E12.5 and E14.5, which was in line with our ISH results (Figure 5A, D). Regarding, *Cxx1c/Mart8a* sense and *Cxx1a,b/Mart8b,c* antisense transcripts only a vague ~1.0 kb transcript was observed at E14.5, supporting an increase of expression after E12.5, similar to ISH (Figure 5A, D). We analyzed *Ldoc1/Mart7* to determine the exact sense and antisense transcript sizes using 5′ and 3′RACE (Figure 7). Using specific primers, which hybridized to the middle of the gene, we identified with 5′ RACE a ~1.0 kb sense transcript and a ~1.4 kb sense transcript using 3′ RACE, which resulted in a total of ~2.4 kb sense transcript for *Ldoc1/Mart7* (Figure 7A). A similar strategy was used to identify the antisense transcript for *Ldoc1/Mart7*. Results demonstrated a ~2.0 kb (5′ RACE) and ~1.0 kb (3′ RACE) antisense transcript totaling ~3.0 kb (Figure 7B). In silico analysis of Ldoc1/Mart7 (NC_000086.7) detected a poly-A consensus site for both the sense (0.97 kb starting from the primer) and antisense (1.32 kb 3′ of the primer) transcripts supporting the 3′RACE results.

### The two highly expressed *Ldoc1/Mart7* and *Cxx1a,b,c/Mart8b,c,a* genes demonstrated differential protein expression throughout placental development

**Figure 8:**
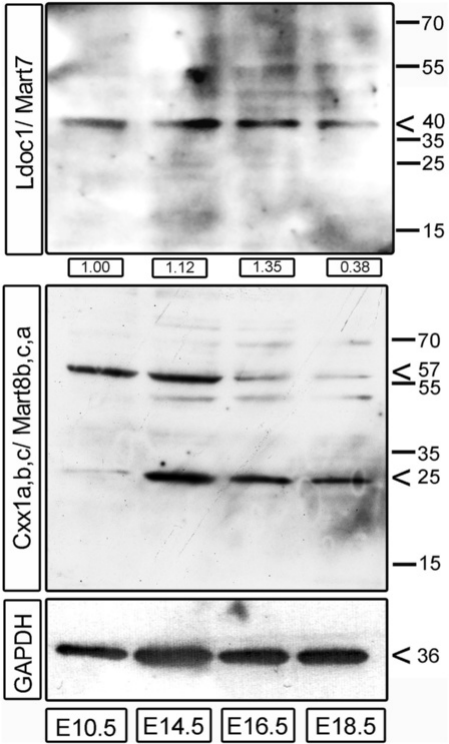
**Protein expression of mouse Ldoc1/Mart7 and Cxx1/ Mart8 in placentae.** Protein lysates of mouse placentae at E10.5, E14.5, E16.5 and E18 were transferred onto nitrocellulose membranes and then hybridized with antibodies specific for Ldoc1/Mart7 and Cxx1/Mart8. A GAPDH immunoblot was used as a normalization control. Numbers on the right site indicates protein sizes in kilodaltons. For Ldoc1/Mart7 semi quantitative protein values normalized to GAPDH are indicated in fold difference below each lane using ImageJ.

To prove the presence of Mart proteins in the mouse placenta Immunoblotting was performed for the two highest *Mart* genes, *Ldoc1/Mart7* and *Cxx1a,b,c/Mart8b,c,a* using protein placental lysates of different developmental stages. Ldoc1/Mart7 has a calculated molecular weight of 17.54 kDa and Cxx1a,b,c/Mart8,b,c,a a mean molecular weight of 13.6 kDa. However, using denaturing SDS-gels and immunoblotting, we detected a prominent band for Ldoc1/Mart7 with a size of approximately ~40 kDa at E10.5, which decreased 2.6-fold in expression at E18.5 (Figure 8). Detection of Cxx1a,b,c/Mart8b,c,a showed a ~57 kDa and a ~25 kDa protein between E10.5 and E18.5. The higher ~57 kDa molecular weight protein was stronger at the earlier stages E10.5 and E14.5, and concurrent with its disappearance, the ~25 kDa protein increased at E16.5 and E18.5.

## Discussion

Transposable elements are of major importance in the mouse genome and research is ongoing to further characterize these genes and define their functions. The most comprehensively examined gene family is the evolutionary conserved group of endogenous retroviruses, which have essential functional roles for their host, e.g. cell fusion during placentogenesis [37]. This report characterized a family of *sushi* retrotransposons, the *Mart* genes, throughout development of the placenta from C57BL/6 and C3H mice, where both strains showed no differences in expression or localization. Implementing quantitative expression analysis, identification and co-localization of *Mart* sense and antisense transcripts, our findings support gene regulatory roles for *Marts* in mouse placentogenesis.

The overall structure and phylogenetic analysis shown in Figure 1 supports the hypothesis that all 11 *Mart* genes evolved from ancestral Metaviridae related to the *sushi-ichi* gene and became diversified by gene duplications [2]. For example, an alignment of *Peg10/Mart2* with *sushi-ichi* (gag) identified 51% DNA nt identity (672 of 1298 nt) and 24% amino acid identity (98 of 406 amino acids) [11]. Pol-like sequences have only been identified in *Rtl1/Mart1*, *Peg10/Mart2* and *Zcchc5/Mart3* [11]. Furthermore, the phylogenetic tree shows relationships between different mouse *Mart* genes, most likely by recombination events including gene duplications, deletions or coding sequence modification. Thus, these processes contributed to their overall gene/protein sizes. For example, protein size variations span from 113 amino acids for Cxx1a,b,c/Mart8b,c,a to 1364 amino acids for Rgag1/Mart9 [11]. Mostly, genes evolve into inactive pseudogenes due to the lack of a promoter. Since a loss of the 5′ and 3′ LTRs of *Mart* genes occurred, their establishment in the genome must have evolved by acquiring new promoter sequences in order to develop into neofunctional genes [38,39]. Different scenarios could have arisen, either the recruitment of distant promoters from other genes or de novo promoter formation [2]. Taken together, *Mart* genes acquired promoters and regulatory elements as well as other structural changes throughout evolution leading to their essential roles in the development of the placenta in mammals, e.g. for *Rtl1/Mart1* and *Peg10/Mart2* [17,22].

The existence of 11 *Mart* genes in mice, and the determination that *Rtl1/Mart1, Peg10/Mart2* and recently *Ldoc1/Mart7* play a functional role during placentogenesis, we focused our study on the expression patterns of Marts not yet described in mouse placental development. Our results showed that not all *Mart* genes were expressed during placentogenesis. Specifically, *Zcchc5/Mart3*, *Zcchc16/Mart4* and *Rgag1/Mart9* were not expressed at the blastocyst (E4.5) stage nor at later stages of placentation, thus we conclude that these three genes play no essential role in placental developmental although a functional role during embryogenesis is possible. On the other hand we found very low expression for the other *Mart* genes in the blastocyst in contrast to higher levels of expression at E8.5 and the following stages, supporting potential functional roles in placental development.

As previously shown, *Rtl1/Mart1* and *Peg10/Mart2* were expressed in mouse placenta with an essential role for embryo survival [17,21,22]. When we compared gene expression of all *Mart* members in this report with our previous study of *Peg10/Mart2* and other developmental genes [21] using the same mouse placentas, *Peg10/Mart2* was the highest expressed retrotransposon gene with a maximum of 188,917.13 molecules/ng cDNA at E16.5 [21]. In contrast, the *Rgag4/Mart5* and *Ldoc1l/Mart6* genes were more constantly expressed at lower levels (between 1.82 and 20.18 molecules/ng cDNA) throughout development (Figure 2B). Furthermore localization of *Rgag4/Mart5* and *Ldoc1l/Mart6* transcripts to trophoblast cells within the junctional zone showed no difference between PAS positive ST and GlyT. This was similar to other placental marker genes, like the *trophoblast specific protein alpha* (*Tpbpα*) and -*beta* (*Tpbpβ*) which also showed a comparable expression pattern between ST and GlyT [21]. The expression similarity noted between these two cell types could result from the fact that GlyT are derived from ST [26] thus both cell types stem from the same progenitor cell. Additionally we showed that *Rgag4/Mart5* and *Ldoc1l/Mart6* intensely localized to the cytosol of pTGCs in the junctional zone, which points to further specialized functions of these *Marts* (Figure 3B). Finally, we revealed antisense expression for *Rgag4/Mart5* and *Ldoc1l/Mart6* using ISH*,* where *Rgag4/Mart5* was stronger at E8.5 compared to *Ldoc1l/Mart6* with a punctate pattern in nuclei. At E14.5 antisense nuclear expression became equally strong for both *Rgag4/Mart5* and *Ldoc1l/Mart6*. Importantly, we found similar ratios of antisense to sense transcripts for *Rgag4/Mart5 and Ldoc1l/Mart6* at E14.5 using TASA-TD. In the literature nuclear localization of antisense RNA transcripts was previously described for other genes [40]. The presence of *Rtl1/Mart1* antisense transcripts was also shown before in mouse and sheep using strand specific PCR and Northern analysis [9,41-43]. Our findings that *Rgag4/Mart5* and *Ldoc1l/Mart6* antisense transcripts were prominent throughout placental development and co-localized to the nuclei support the idea that sense transcription may be linked with antisense regulation.

*Ldoc1/Mart7* and *Cxx1a,b,c/Mart8b,c,a* RNA expression was high during placentogenesis and also showed strong sense and antisense expression localizing to STs, GlyTs and pTGCs especially at later stages. A very recent analysis of *Ldoc1/Mart7* knockout mice showed a disturbed placental endocrine function (overproduction of progesterone) and delayed parturition (1–4 days later) [35]. The latter analysis of *Ldoc1/Mart7* localization by ISH along with decreased numbers of ST in the placentae of the knockout mice confirmed our localization results along with the functional importance of the gene in pTGC, GlyT and especially ST. In addition to *Cxx1a,b,c/Mart8b,c,a* expression in the junctional zone, positive expression was also noted in GlyT islands in the labyrinth layer. Similar to *Rgag4/Mart5* and *Ldoc1l/Mart6* strong nuclear expression in the pTGCs was found for *Cxx1c/Mart8a* antisense and *Cxx1a,b/Mart8b,c* sense at E8.5 using ISH. Due to the high homology and gene orientation of the *Cxx1a,b,c/Mart8b,c,a* gene cluster, RNA probes cross-hybridized thus we could not identify single independent transcripts using ISH. Therefore for verification of the antisense and sense *Mart* transcripts by ISH, we developed a novel method TASA-TD to specifically identify and quantify sense and antisense transcripts in tissues. Based upon a specific first strand cDNA synthesis of isolated RNA and using a non-murine TAG-sequence, we detected sense and antisense transcripts of *Rtl1/Mart1, Rgag4/Mart5, Ldoc1l/Mart6, Ldoc1/Mart7, Cxx1c/Mart8a and Cxx1a,b/Mart8b,c* in E14.5 placenta. If antisense transcripts regulate transcription and esp. translation of sense transcripts, *Rtl1/Mart1* and *Cxx1a,b,c/Mart8b,c,a* are more highly regulated than *Rgag4/Mart5, Ldoc1l/Mart6* and *Ldoc1/Mart7* due to their higher ratios of antisense: sense transcripts. Furthermore, using Northern blot analysis of *Cxx1a,b,c/Mart8b,c,a* and 5′ and 3′ RACE methodology of *Ldoc1/Mart7* determined sense and antisense transcript sizes corroborating both TASA-TD results and ISH expression.

Thus, we can conclude that expression of the *Rgag4/Mart5 to Cxx1a,b,c/Mart8b,c,a* genes are mainly restricted to trophoblast cells in the junctional zone. This was distinctly different to *Rtl1/Mart1* and *Peg10/Mart2,* where *Rtl1/Mart1* only localized in fetal capillaries of the labyrinth [17] and *Peg10/Mart2,* in addition to expression in the junctional zone, localized specifically to sTGCs of the labyrinth layer, supporting functional roles at the fetal-maternal blood barrier [21,22]. Furthermore, siRNA studies comparing *Peg10/Mart2* with the fusogenic ERV genes, *Syncytin-A* and *Syncytin-B*, which were restricted to SCT-I and –II in the labyrinth, demonstrated no functional role of *Peg10/Mart2* in cell fusion [21]. Therefore, we predict that the *Marts* expressed in different trophoblast cells have no functional roles in cell fusion [28].

There are multiple mechanisms by which antisense transcripts regulate sense transcription leading to changes in gene/protein expression. These include, direct inhibition of sense transcription via antisense hybridization to RNA; induction of DNA methylation for gene silencing by antisense mediation; blockage of RNA splicing, processing, stability and miRNA binding sites via antisense/sense hybrids; DICER targeting and processing of antisense/sense hybrids to siRNAs; induction of RNA editing or changes of secondary structures through antisense/sense hybrids and finally, inhibition of translation due to cytoplasmic antisense/sense hybrids [44-47]. The presence of antisense transcripts of imprinted genes and their regulatory role has been shown previously [48,49]. *Rtl1/Mart1* (*Peg11*) maternally expressed antisense transcripts code for miRNAs and target the *Rtl1/Mart1* transcript [42]. Therefore, fine tuning of gene expression is possible by miRNAs, which are imbedded within antisense transcripts. An example of transposon silencing by RNAi was found in germ line cells of *Caenorhabditis elegans* and was directly linked with transposon antisense RNA during development [50]. Antisense transcripts leading to gene silencing and methylation as well as to defects in transcriptional regulation can also result in diseases [51]. For example an antisense transcript specific for the *β-site APP-cleaving enzyme 1* gene (*BACE1*) encodes for an enzyme which has an important role in progression of Alzheimer’s disease [52].

Verification of protein supports regulation beyond the cellular functions of RNA. Comparing Ldoc1/Mart7 and Cxx1/Mart8 proteins we found regulatory differences throughout placental development. By E18.5 the ~40 kDa Ldoc1/Mart7 protein was decreased by 2.6-fold compared to E10.5, which corroborated our qPCR data. On the other hand, Cxx1a,b,c/Mart8b,c,a showed a unique developmental regulation such that a ~57 kDa protein was detected in earlier stages of placentation but in later stages a ~25 kDa protein was found. It should be noted that the calculated protein size for Ldoc1/Mart7 is 17.54 kDa (IEP: 4.08) and for Cxx1a,b,c/Mart8b,c,a 13.6 kDa (IEP: 9.02). However, the observed protein sizes with repeated SDS-PAGE/ Immunoblots were ~40 kDa for Ldoc1/Mart7 and for Cxx1/Mart8 ~ 25 and ~57 kDa (Figure 8). These discrepancies between the calculated and our observed protein sizes for both Ldoc1/Mart7 and Cxx1/Mart8 are too big to be explained by post-translational modifications. Considering the different protein sizes in immunoblots after SDS-PAGE several other explanations could be possible: 1) Proteins with multiple hydrophobic residues can load more SDS and change the PAGE mobility [53]. For example, comparing the hydrophobicity index of −0.12 for the reference protein GAPDH (calculated 38.64 kDa, IEP: 9.6) with the hydrophobicity indexes of Ldoc1/Mart7 at −0.39 and Cxx1a/Mart8b at −0.7, calculated using GPMAW [54], it is possible that the higher hydrophobicity of Ldoc1/Mart7 and Cxx1/Mart8 could shift the SDS-PAGE mobility; and 2) Dimerization and oligomerization of proteins could have occurred during SDS-PAGE and Immunoblotting. Although SDS normally prevents stable protein-protein associations, SDS can also induce protein dimerization [55]. Importantly were the observations by Rey et al. [56] that protein-protein dimers were found stable for env proteins of HIV-2 and SIV after SDS treatment. Due to the fact that Mart proteins are gag-like proteins and it is known that gag proteins of HIV-1 form protein dimers [57], even after SDS-treatment of murine sarcoma virus gag proteins [58], it is conceivable that Ldoc1/Mart7 and Cxx1/Mart8 exist as different protein oligomer species. Thus, for our Immunoblotting results in Figure 8, we predict that Ldoc1/Mart7 represents protein dimers of 17.54 kDa × 2 = 35.08 kDa (observed ~40 kDa in immunoblots). For Cxx1/Mart8 we predict dimers at E14.5 to E18.5 (13.6 kDa × 2 = 27.2 kDa; observed ~25 kDa) and protein tetramers (13.6 kDa × 4 = 54.4 kDa; observed ~57 kDa) at E10.5 and E14.5. If these protein dimers and tetramers were induced by SDS or occur *in vivo* has to be further analyzed.

Although, protein expression of Rtl1/Mart1 and Peg10/Mart2 was demonstrated as essential for placental development [17,22,52], functions of other *Mart* genes in the mouse placenta are still unknown. Mart protein domains shown in Figure 1 suggest a variety of roles in cellular processes. For example Peg10/Mart2 was also described as a zinc-finger transcription factor regulating myelin protein expression in murine brain development [5]. Other functional analyses have been demonstrated in context with human *MART* genes. One study showed the human PEG10/MART2-ORF2 protein binding to the TGF-beta receptor ALK1 (activin receptor-like kinase 1), which resulted in receptor inhibition. Co-expression of both proteins in cell-lines led to morphological changes [59]. Okabe et al. demonstrated PEG10/MART2 overexpression in hepatocellular carcinomas with a role in inhibition of apoptosis [60]. In contrast, LDOC1/MART7 showed down regulation of expression in carcinoma cells, supporting possible tumor suppressor activity [8]. Therefore, it is possible that most of the *MART* genes, like *RTL1/MART1, PEG10/MART2, LDOC1L/MART6, LDOC1/MART7* and *RGAG1/MART9* may play a role in tumorigenesis [61,62].

Taken together, our findings that *Mart* gene and protein expression occur throughout mouse placental development speaks for essential functions during placentogenesis.

## Conclusion

Our results confirm the hypothesis of neofunctionalization of retroelements in mammals throughout evolution and the conservation of their cellular functions for placental development and ultimately, offspring survival. Eleven mouse *Mart* genes derived from the *gag* genes of Ty3/gypsy LTR retroelements showed different expression patterns in mouse placentation. *Rgag4/Mart5 to Cxx1a,b,c/Mart8b,c,a* gene expression in the mouse placenta demonstrated specific localization in trophoblast lineages, which evolved from cells of the ectoplacental cone. Presence of antisense transcripts and alterations in protein expression at different developmental stages points to complex regulatory mechanisms of sense transcript and protein expression for the *Marts*. We predict that due to the similar homology between mouse *Mart* and human *MART* genes their functional roles could be analogous during placental development.

## Methods

### Mice and placenta preparation

Pregnant C57BL/6 and C3H mice were provided from the Institute of Biochemistry of Friedrich-Alexander-University Erlangen-Nürnberg. Experiments were performed in strict accordance with the protocol, which was approved by the Committee on the Ethics of Animal Experiments of the University of Erlangen-Nürnberg (Permit Number: TS-00/12- Biochemistry II). Placentae for RNA isolation and frozen sections were prepared according to previously published methods [21]. Blastocyts from C57BL/6 mice were provided by Dr. Megan Mitchell, University-Clinic, Department of Gynaecology and Obstetrics, Erlangen.

### Phylogenetic analyses

A multiple alignment, phylogenetic reconstruction and graphical representation for all 11 mouse *Mart* genes was performed according to Dereeper et al. [63] using http://www.phylogeny.fr/. For this analysis the entire codogenic sequence of every mouse *Mart* gene was used according to NCBI references: *Rtl1/Mart1* (NM_184109.1), *Peg10/Mart2* (NM_001040611.1), *Zcchc3/Mart3* (NM_199468.1), *Zcchc16/Mart4* (NM_001033795.4); *Rgag4/Mart5* (NM_001278534.1), *Ldoc1l/Mart6* (NM_177630.3), *Ldoc1/Mart7* (NM_001018087.1), *Cxx1a/Mart8b* (NM_024170.2), *Cxx1b/Mart8c* (NM_001018063.1), *Cxx1c/Mart8a* (NM_028375.3) and *Rgag1/Mart9* (NM_001040434.2).

### Periodic acid-Schiff stain

Paraffin embedded mouse placenta of E15.5 were cut into 2 μm tissue sections using a microtome (Microm, Heidelberg), de-paraffinized, rehydrated and treated with 1% periodic acid (wt/vol) (Sigma, Germany) in water for 5 min at room temperature according to Tunster et al. [64].

### RNA extraction and cDNA synthesis

Total RNA from mouse snap frozen placentae was extracted using peqGOLD TriFast (PEQLAB Technologies) according to manufactures’ protocol. After precipitation the extracted RNA was solubilized in 0.1% DEPC-treated water and then pre-treated with 40 U DNaseI (Roche, Germany) for 60 min at 37°C. RNA from 10 and 17 pooled blastocysts (E4.5) was extracted using the Absolute RNA Nanoprep Kit (Agilent, Germany) according to the company’s protocol then cDNA was synthesized using the High Capacity cDNA Kit (Applied Biosystems (ABI), Germany) in a thermal cycler (Applied Biosystems, Germany) for 2 h at 37°C.

### Absolute quantitative real time PCR (qPCR)

For qPCR, gene fragments of interest were amplified with specific primers (Additional file 1: Table S1) and cloned directly via Topoisomerase I bound vector arms (PCR insertion site) into a pSC-A vector (Stratagene, Germany). From each cloned *Mart* gene the copy numbers were calculated and used as an external standard to generate a standard curve with a cycle threshold (Ct) value against the log of amount of standard. Expression values were calculated as molecules per ng total cDNA using a standard curve of each cloned *Mart* gene determined by real time PCR. The efficiencies (γ) of the qPCR were between −3.52 to −3.17, the limit of detection (t) and the correlation coefficient (R^2^) is documented in Additional file 2: Table S2. For gene quantification of the blastocyst state and placentae from stage E8.5 to E18.5, SYBR-green (Thermo Fisher, Germany) based qPCR with specific Primers (Additional file 1: Table S1) and 40 ng cDNA per well (4 ng cDNA per well for blastocyst) were used with an ABI7300. The amplicon sizes of the Mart genes were between 90 and 127 bp. 18SrRNA amplification of the probes using 1 ng cDNA was used for normalization (primer Additional file 1: Table S1) and one probe was used in every qPCR as internal control. All expression values in molecules/ ng cDNA are shown (Additional file 3: Table S3).

### *In situ* hybridization (ISH)

For the synthesis of the specific digoxigenin (DIG)-labeled sense and antisense RNA probes of *Rgag4/Mart5*, *Ldoc1l/Mart6*, *Ldoc1/Mart7* and *Cxx1a/Mart8b* plasmids were linearized with restriction enzymes. *In vitro* transcription and DIG-labeling was performed with a RNA-Labelling Kit (Roche, Germany). All probes were then pretreated with 40 U DNaseI (Roche, Germany) for 60 min at 37°C. Tissue preparation and ISH of mouse placentae of different embryonic stages were performed according to published methods [21]. A control ISH without sense or antisense RNA probe was performed for the evaluation of artifactual background signals (Additional file 4: Figure S1).

### PCR analysis

*Cxx1a,b,c/Mart8b,c,a* expression was analyzed via PCR with specific primers (Additional file 1: Table S1) and 100 ng cDNA generated from E8.5, E12.5, E14.5, E16.5 and E18.5 placental RNA probes. PCR reactions were implemented with the Fast Start Taq-Polymerase Kit (Roche, Germany). Amplified fragments were visualized on a 1% agarose gel with ethidium bromide staining.

### Northern Blot of *Cxx1a,b,c/ Mart8b,c,a*

Four μg DNase I digested placental RNA (E12.5 and E14.5) was denatured in a buffer with: 1 × MOPS, 50% formamide, 20% formaldehyde for 10 min at 65°C and electrophoresed in a 1.2% agarose gel containing 2.2 M formaldehyde and 1 × MOPS. The transfer was done overnight by capillary blot methodology onto a nylon membrane in the presence of 20 × SSC. After transfer, the RNA was fixed at 80°C for 2 h. Prehybridization was done with ULTRAhyb® Ultrasensitive Hybridization Buffer (Ambion/Applied Biosystems) at 68°C for 1 h. To detect both transcripts, blots were hybridized separately with antisense or sense Cxx1a/Mart8b DIG-labeled nucleotide probes at a concentration of 100 ng/ml overnight at 68°C. After stringency washes with 2 × SSC/ 0.1% SDS, 1 × SSC/ 0.1% SDS and 0.5 × SSC/ 0.1% SDS the membranes were incubated with a Blocking Buffer (Sigma) for 30 min. Detection of RNA-probe hybrids was performed with anti-digoxigenin-AP, Fab-fragments (1: 10,000; Roche) and a chemi-luminescence reaction with AP-Juice (PJK, Germany), and then visualized with X-ray films.

### 5′ and 3′ rapid amplification of cDNA ends (RACE) of *Ldoc1/ Mart7*

The 5′/ 3′ RACE for *Ldoc1/Mart7* sense and antisense transcripts was performed with the 5′/3′ RACE Kit, 2nd Generation (Roche) according to the manufactures protocol. Briefly, 200 ng placental RNA (E14.5) was used for the *Ldoc1/Mart7* sense transcript with a gene specific (GS) sense (s) primer (GS-s-cDNA) for the 5′RACE first strand cDNA synthesis. After purification of the cDNA with the High Pure PCR product purification kit (Roche), a homo-polymeric d(A)-tail was ligated to the 3′end of the first strand cDNA using a terminal transferase and dATP. PCR amplification of poly-d(A)-tailed cDNA was performed with an oligo-d(T)-anchor primer and a GS-s-BR-PCR primer. For the 3′RACE of *Ldoc1/ Mart7* sense transcript, 500 ng placental RNA (E14.5) was transcribed with an oligo-d(T)-anchor primer in the first strand cDNA. PCR amplification was done with a GS-s-TF primer and a BR anchor primer (Additional file 1: Table S1). In order to analyze *Ldoc1/Mart7* antisense transcripts, antisense specific primers were used. For the 5′ RACE of the antisense transcripts a GS-as-cDNA primer for cDNA synthesis and GS-as-BR-PCR primer for amplification were implemented. To analyze the 3′end of the *Ldoc1/Mart7* antisense transcript, poly(A)-tailing was performed at the 3′end of placental RNA (E14.5) with a poly(A)-polymerase (New England Biolabs). For the first strand cDNA synthesis 500 ng of the poly(A)-tailed RNA was used with an oligo-d(T)-anchor primer and gene specific amplification followed with a GS-as-TF primer and an BR anchor primer (Additional file 1: Table S1). Q5®-High-Fidelity DNA-polymerase (New England Biolabs) was utilized for all amplification PCRs. Amplified fragments were visualized on a 1% agarose gel with ethidium bromide staining. Lengths of the transcripts were calculated according to a 100 bp and a 1 kb DNA ladder (Bio&Sell).

### First strand cDNA synthesis and strand specific PCR

#### Overview

We developed the following new approach called the TAG-aided sense/antisense transcript detection (TASA-TD) method in order to identify and quantify sense and antisense transcripts. For sense and antisense RNA transcript analysis RNA from E14.5 placenta was isolated as described above. An overview of the technique is represented in Figure 6A. In order to amplify and primer extend the specific gene of interest from independent sense or antisense transcripts the first step involved annealing a gene specific primer (GSP) fused to a TAG-sequence not specific for the mouse genome (GSP sense/antisense (RT) TAG). The resulting single sense or antisense cDNA/RNA hybrids were then digested with RNase H to generate single strand cDNAs and then further amplified using a 5′ → 3′ GSP (PCR) and the 3′ → 5′ TAG primer (Figure 6A). All cDNA products were electrophoresed on 1% agarose gels, visualized using ethidium bromide and then original Tif-images quantified using ImageJ (http://imagej.nih.gov).

#### Specific methodology

Primer sequences are shown in Additional file 1: Table S1C. Specific components from the SuperScript III First-Strand Synthesis System for RT-PCR (Life technologies, Germany) were implemented and adapted for our methodology to perform reverse transcription from placental RNA. For the first strand cDNA synthesis reaction 50 ng RNA for *β-actin* and *Ldoc1/Mart7*, 400 ng RNA for *Rgag4/Mart5* and *Ldoc1l/Mart6* and 100 ng for *Rtl1/Mart1* and *Cxx1c/Mart8a* and *Cxx1a,b/Mart8b,c* was used. Furthermore 1 μM GSP-TAG, 0.5 mM dNTP, 5 mM MgCl_2_, 10 mM DTT, 40 U RNaseOUT, 100 U SuperScriptIII® RT (life technologies, Germany) and 240 ng Actinomycin D (Sigma, Germany) were added for a 20 μl reaction. Importantly, a RT with very low intrinsic RNase H activity (for cleavage of RNA from RNA/DNA duplexes) and Actinomycin D was necessary to prevent RT of second strand cDNA and thus antisense artifacts [36]. RNA and primers were preheated at 65°C for 5 min. Synthesis was performed at 50°C for 50 min and terminated at 85°C for 5 min. After cDNA synthesis 2 U recombinant RNase H (life technologies) was added to each reaction and incubated 20 min at 37°C. The first strand cDNA mix was then purified via ethanol precipitation and dissolved in 10 μl sterile water. Afterwards gene and strand specific PCR was performed. To amplify sense cDNA a TAG-primer and GSP sense (PCR) were used. Amplification of antisense cDNA was performed with the TAG-primer and the GSP antisense (PCR). As an internal negative control we performed sense and antisense specific PCR for both sense and antisense cDNA of *β-actin* which was shown to have no antisense transcript [47]. PCR reactions were implemented with the Fast-Start Taq-Polymerase Kit (Roche) as described above (PCR analysis).

### Immunoblotting

Mart7 and Mart8 protein expression was analyzed in lysates from mouse placentae of stage E10.5, E14.5, E16.5 and E18.5 according to Strick et al. [65]. Fifteen micrograms of the lysates were resolved on a 7.5% - 12.5% acrylamide gradient SDS-gel, transferred to a nitrocellulose membrane using a CAPS buffer [65]. After blocking with 5% BSA (Sigma-Aldrich, Germany) (Ldoc1/Mart7, Cxx1/Mart8) or Blocking Buffer (Sigma-Aldrich, Germany) for GAPDH, membranes were hybridized with specific antibodies (polyclonal rabbit anti mouse LDOC1, Biozol, Germany, 1: 800; polyclonal rabbit anti mouse CXX1, Bioss, USA 1: 800). A secondary peroxidase labeled antibody was used for detection (goat anti rabbit HRP, Cell Signaling, Germany, 1: 1,000). After Ldoc1/Mart7 or Cxx1/Mart8 protein detection, membranes were first washed 5 min in TBST and then incubated 5 min in a stripping buffer (Thermo Scientific) at room temperature. Afterwards, the membranes were washed in TBS for 5 min and then incubated with Blocking Buffer (Sigma) for 30 min at room temperature. For normalization detection of the control protein GAPDH was used (polyclonal rabbit anti mouse GAPDH-HRP, Santa Cruz, Germany, 1 : 1,000) and hybridized to previously stripped membranes. Protein expression was detected using a chemiluminescence reaction with HRP-Juice (PJK, Germany) then visualized with X-ray film. Ldoc1/Mart7 protein was normalized to GAPDH and quantified using ImageJ (http://imagej.nih.gov).

## Additional files

Additional file 1: Table S1. Primers. Additional file 2: Table S2. Standard curves. Additional file 3: Table S3. Mart absolute gene expression values. Gene expression of murine Mart genes in placentae during embryonic development from E8.5 to E18.5; n= different placentae. Additional file 4:
**In situ hybridization (ISH) negative control.** ISH was done according to the method described using placental sections from E15.5, but without any sense or antisense Mart RNA probe. Nuclei were stained with nuclear fast red. Panels show two magnifications of cells of the decidua (d) junctional zone (jz) with parietal trophoblast giant cells (pTGC) (arrow) and the labyrinth layer (l).
