# Supplementary material for: Selective expression of sense and antisense transcripts of the sushi-ichi-related retrotransposon – derived family during mouse placentogenesis
Source: Retrovirology. 2015 Feb 3;12:9. doi: 10.1186/s12977-015-0138-8 (PMC4340606; doi:10.1186/s12977-015-0138-8)
Supplement: Additional file 1: Table S1. — Primers. [file 12977_2015_138_MOESM1_ESM.docx]

Additional file 1: Table S1: Primers.

| **A: Primer for quantitative real time PCR** | |  |
| --- | --- | --- |
| **Primer** | **Sequences for cloning (5´-3´)** | **Sequences for real time PCR (5´-3´)** |
| 18SrRNA TF |  | ATGGCCCTTAGTTGGTG |
| 18SrRNA BR |  | GAACGCCACTTGTCCCTCTA |
| Mart1 TF | AGAAAGCTTATGATAGAACCCTCTGAAGACTCATTTGA | GAAACAATCAACTCATCCGAGAC |
| Mart1 BR | TCAGTCAACTTCATCATCTGAGTCTGATGGATCCTCT | AGAGTTCTTGGGCTGACCTTC |
| Mart2 TF | ATGGCTGCTGCGGGTGGTTCCTCCAACTGC | ACATGAACAGGCAGTTTGAGAATA |
| Mart2 BR | CTACAGCGGGGCCGGGGAGTTTCCCGGCGGC | TCTTCATCATCATCAAAGTCA |
| Mart3 TF | CTAGAACTCTCAGTACCTGAAGAGT | TCAGTTGACCAGTTACCTGAGAAG |
| Mart3 BR | GCCTGCTGGACACGATGAGGCTTGACA | TATCTAATAAGGGCTGGAACATCC |
| Mart4 TF | ATGGAGAAGTGTACAGAATCTCTACCA | TGAGGGCTCTGGATATTTCTAATC |
| Mart4 BR | TCTAGCTGAATGCACTGAGTGATC | TGGGTAGACTCAGATAGGACATCA |
| Mart5 TF | TGATGCAGCTAGAGACTTTCATAGCAGA | GCTATGCAGATGCTTTTCAGTTC |
| Mart5 BR | CTGTCATGTGCTGCCTCTGATCCTTCAG | GCCACTTCAGTTGACCATAAGAG |
| Mart6 TF | ATGGTCCAACCTCGGACCTCCAAAACTG | TTACTCCACCTATCTCTGCCATC |
| Mart6 BR | TTAAAGGTTCCGGCCACGAGAGGGCAAGGCT | GGTCTCCTGAGAAGGGTTCAG |
| Mart7 TF | ATGGTGGAAGAGCTGCTGTCGCTTCTGC | CATTCTTGATCAGCCTCCTCTC |
| Mart7 BR | CTAGTGATCTTCCTCCATTTCTTCCTCTTCA | ATCTCATCAACAAAGGCCTGATA |
| Mart8 TF | CTCCCGGAGTTCATCGTCCAGACC | AAGGGCCGGGCCCTGCAGTG |
| Mart8 BR | CTAGAAGTCCTCATCCTCCTCCCACCCG | CTAGAAGTCCTCATCCTCCTCCCACCCG |
| Mart9 TF | AGCAATGCCTACACCACTCCTGTCAAT | CACCAATAAGAGTTTCATCCACTG |
| Mart9 BR | ATCTGTGGCACGGATATCACTCCTGA | CTCTCATTAGCTGTATGGGTGTTG |

Mart1 was cloned with a Hind III (Mart1 TF) and Bam H1 (Mart1 BR) linker. Mart2 was the N-terminal part cloned.

| **B: Primer for Mart8 specific PCR** | |
| --- | --- |
| **Primer** | **Sequences for PCR (5´-3´)** |
| Mart8a TF | CCATGGACCGCCGGATTAAGTTGATT |
| Mart8a BR | GTAGCAGCTCACCCTCTTGTAGCCCCTG |
| Mart8bc TF | AGGCCAAGGCAAGGTAAAGAGGCCGAA |
| Mart8bc BR | AGCAGCCCACCCTCCCGGAGCCCCAA |
| β-actin TF | GCTGGTCGTCGACAACGGCTC |
| β-actin BR | CAAACATGATCTGGGTCATCTTTTC |

| **C: TAG labeled Primer for first strand cDNA Synthesis and gene and strand specific PCR** | |
| --- | --- |
| **Primer** | **Sequences for first strand cDNA synthesis (GSP sense/antisense TAG) (5´-3´)** |
| β-actin sense TAG | **GCACACGACGACAGACGACGCAC**CAAACATGATCTGGGTCATCTTTTC |
| β-actin antisense TAG | **GCACACGACGACAGACGACGCAC**GCTGGTCGTCGACAACGGCTCCGGCAT |
| Mart1 sense TAG | **GCACACGACGACAGACGACGCAC**GATCCTCTGTGTTGAGAAGGATCATG |
| Mart1 antisense TAG | **GCACACGACGACAGACGACGCAC**TGGCAGAGAGGTCCTGGTCTACTCAA |
| Mart5 sense TAG | **GCACACGACGACAGACGACGCAC**CTGTCATGTGCTGCCTCTGATCCTTC |
| Mart5 antisense TAG | **GCACACGACGACAGACGACGCAC**TGATGCAGCTAGAGACTTTCATAGCA |
| Mart6 sense TAG | **GCACACGACGACAGACGACGCAC**ATACCAGGAAGGCCACTCTCTCGGCT |
| Mart6 antisense TAG | **GCACACGACGACAGACGACGCAC**ATGGTCCAACCTCGGACCTCCAAAAC |
| Mart7 sense TAG | **GCACACGACGACAGACGACGCAC**CTAGTGATCTTCCTCCATTTCTTCCTC |
| Mart7 antisense TAG | **GCACACGACGACAGACGACGCAC**ATGGTGGAAGAGCTGCTGTCGCTTC |
| Mart8a sense TAG | **GCACACGACGACAGACGACGCAC**GTAGCAGCTCACCCTCTTGTAGCC |
| Mart8a antisense TAG | **GCACACGACGACAGACGACGCAC**CCATGGACCGCCGGATTAAGTTGAT |
| Mart8bc sense TAG | **GCACACGACGACAGACGACGCAC**AGCAGCCCACCCTCCCGGAGCCCCAA |
| Mart8bc antisense TAG | **GCACACGACGACAGACGACGCAC**AGGCCAAGGCAAGGTAAAGAGGCCGA |
| **Primer** | **Sequences for gene and strand specific PCR (5’-3’)** |
| ß-actin sense | GCTGGTCGTCGACAACGGCTC |
| ß-actin antisense | CAAACATGATCTGGGTCATCTTTTC |
| Mart1 sense | TGGCAGAGAGGTCCTGGTCTACTCAA |
| Mart1 antisense | GATCCTCTGTGTTGAGAAGGATCATG |
| Mart5 sense | TGATGCAGCTAGAGACTTTCATAGCAGA |
| Mart5 antisense | CTGTCATGTGCTGCCTCTGATCCTTCAG |
| Mart6 sense | ATGGTCCAACCTCGGACCTCCAAAACTG |
| Mart6 antisense | ATACCAGGAAGGCCACTCTCTCGGCTTC |
| Mart7 sense | ATGGTGGAAGAGCTGCTGTCGCTTCTGC |
| Mart7 antisense | CTAGTGATCTTCCTCCATTTCTTCCTCTTCA |
| Mart8a sense | CCATGGACCGCCGGATTAAGTTGATT |
| Mart8a antisense | GTAGCAGCTCACCCTCTTGTAGCCCCTG |
| Mart8bc sense | AGGCCAAGGCAAGGTAAAGAGGCCGAA |
| Mart8bc antisense | AGCAGCCCACCCTCCCGGAGCCCCAA |
| TAG-Primer | **GCACACGACGACAGACGACGCAC** |
