# Supplementary material for: Selective expression of sense and antisense transcripts of the sushi-ichi-related retrotransposon – derived family during mouse placentogenesis
Source: Retrovirology. 2015 Feb 3;12:9. doi: 10.1186/s12977-015-0138-8 (PMC4340606; doi:10.1186/s12977-015-0138-8)
Supplement: Additional file 2: Table S2. — Standard curves. [file 12977_2015_138_MOESM2_ESM.docx]

Additional file 2: Table S2: Standard curves

| **gene** | **vector** | **γ** | **t** | **R^2^** |
| --- | --- | --- | --- | --- |
| Mart1 | pSC-A amp/kan | -3.4973 | 34.1400 | 0.9958 |
| Mart2 | pSC-A amp/kan | -3.2074 | 33.0470 | 0.9872 |
| Mart5 | pSC-A amp/kan | -3.2588 | 32.7330 | 0.9931 |
| Mart6 | pSC-A amp/kan | -3.5206 | 34.7485 | 0.9959 |
| Mart7 | pSC-A amp/kan | -3.3015 | 34.8437 | 0.9952 |
| Mart8 | pSC-A amp/kan | -3.1766 | 34.3410 | 0.9917 |
