# Supplementary material for: Selective expression of sense and antisense transcripts of the sushi-ichi-related retrotransposon – derived family during mouse placentogenesis
Source: Retrovirology. 2015 Feb 3;12:9. doi: 10.1186/s12977-015-0138-8 (PMC4340606; doi:10.1186/s12977-015-0138-8)
Supplement: Additional file 3: Table S3. — Mart absolute gene expression values. Gene expression of murine Mart genes in placentae during embryonic development from E8.5 to E18.5; n= different placentae. [file 12977_2015_138_MOESM3_ESM.docx]

Additional file 3: Table S3: Gene expression of murine Mart genes in placentae during embryonic development from E8.5 to E18.5; n= different placentae. - ***** Expression values of Mart2 are taken from Henke et al. for comparison [21].

| **embryonic stage** |  | **Mart1** | **Mart2*** | **Mart5** | **Mart6** | **Mart7** | **Mart8a,b,c** |
| --- | --- | --- | --- | --- | --- | --- | --- |
|  |  | [molecules/ ng cDNA] for mean and ± sem | | | | | |
| **E8.5** | mean | 0.47 | 7,766.46 | 3.93 | 7.01 | 73.50 | 1,061.00 |
|  | ± sem | 0.13 | 1,688.47 | 0.63 | 0.80 | 22.21 | 179.68 |
|  | n= | 4 | 4 | 4 | 4 | 4 | 4 |
| **E9.5** | mean | 2.30 | 22,462.77 | 2.76 | 11.03 | 363.16 | 1,489.33 |
|  | ± sem | 0.51 | 1,464.59 | 0.35 | 1.68 | 57.51 | 197.15 |
|  | n= | 6 | 6 | 6 | 6 | 6 | 6 |
| **E10.5** | mean | 7.55 | 71,486.52 | 2.84 | 10.90 | 567.50 | 1,327.33 |
|  | ± sem | 2.11 | 20,438.21 | 0.23 | 0.68 | 115.45 | 142.65 |
|  | n= | 6 | 6 | 6 | 6 | 6 | 6 |
| **E11.5** | mean | 15.40 | 76,936.49 | 2.94 | 15.27 | 660.00 | 977.66 |
|  | ± sem | 2.66 | 10,692.65 | 0.29 | 2.02 | 62.83 | 141.98 |
|  | n= | 6 | 6 | 6 | 6 | 6 | 6 |
| **E12.5** | mean | 29.07 | 116,037.30 | 3.94 | 15.92 | 737.16 | 1,476.83 |
|  | ± sem | 5.46 | 13,514.85 | 0.72 | 3.59 | 176.91 | 353.13 |
|  | n= | 6 | 6 | 6 | 6 | 6 | 6 |
| **E13.5** | mean | 32.60 | 67,678.75 | 2.86 | 13.59 | 408.85 | 1,080.83 |
|  | ± sem | 8.33 | 14,852.90 | 0.54 | 2.35 | 61.07 | 210.13 |
|  | n= | 7 | 7 | 7 | 7 | 7 | 7 |
| **E14.5** | mean | 64.38 | 75,965.82 | 2.40 | 15.70 | 234.33 | 974.66 |
|  | ± sem | 6.46 | 6,949.10 | 0.23 | 1.52 | 36.09 | 105.95 |
|  | n= | 6 | 6 | 6 | 6 | 6 | 6 |
| **E15.5** | mean | 44.80 | 102,615.03 | 1.82 | 14.59 | 289.80 | 927.40 |
|  | ± sem | 5.39 | 10,513.51 | 0.12 | 2.03 | 44.75 | 85.02 |
|  | n= | 5 | 5 | 5 | 5 | 5 | 5 |
| **E16.5** | mean | 72.23 | 188,917.13 | 4.60 | 20.18 | 208.16 | 1,302.66 |
|  | ± sem | 9.69 | 12,010.21 | 0.73 | 3.11 | 53.71 | 178.41 |
|  | n= | 6 | 5 | 6 | 6 | 6 | 6 |
| **E17.5** | mean | 63.30 | 99,951.00 | 3.10 | 19.12 | 86.33 | 1,370.66 |
|  | ± sem | 6.32 | 6,363.65 | 0.67 | 2.02 | 21.88 | 210.91 |
|  | n= | 6 | 6 | 6 | 6 | 6 | 6 |
| **E18.5** | mean | 60.45 | 102,204.98 | 4.51 | 8.36 | 33.55 | 1,431.50 |
|  | ± sem | 9.25 | 3,581.07 | 1.93 | 2.48 | 9.10 | 368.58 |
|  | n= | 8 | 8 | 8 | 9 | 9 | 8 |
