# Supplementary figures and images for: Selective expression of sense and antisense transcripts of the sushi-ichi-related retrotransposon – derived family during mouse placentogenesis
Source: Retrovirology. 2015 Feb 3;12:9. doi: 10.1186/s12977-015-0138-8 (PMC4340606; doi:10.1186/s12977-015-0138-8)

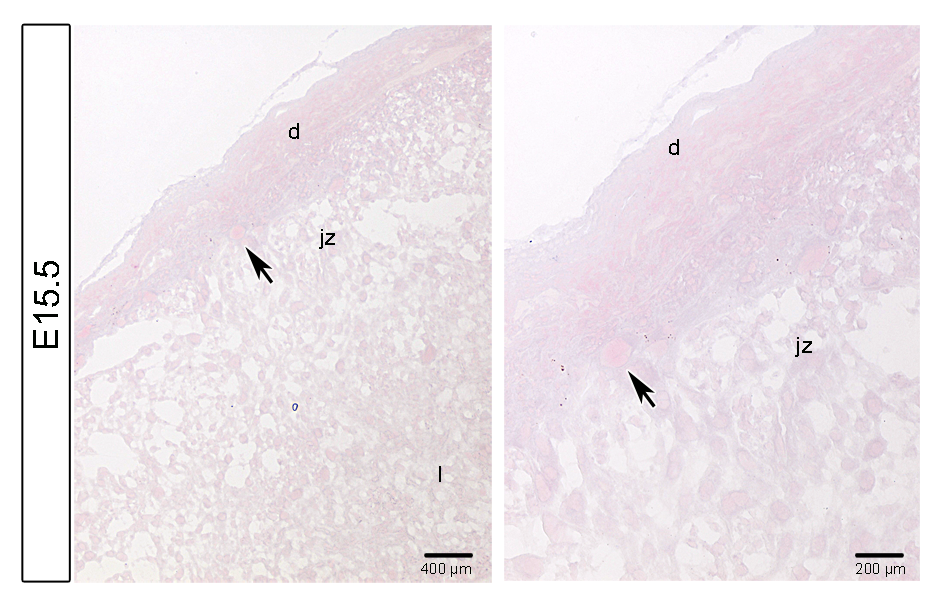

Supplement: Additional file 4: — In situ hybridization (ISH) negative control. ISH was done according to the method described using placental sections from E15.5, but without any sense or antisense Mart RNA probe. Nuclei were stained with nuclear fast red. Panels show two magnifications of cells of the decidua (d) junctional zone (jz) with parietal trophoblast giant cells (pTGC) (arrow) and the labyrinth layer (l). [file 12977_2015_138_MOESM4_ESM.tif]
